# Inducing Sadness and Anxiousness through Visual Media: Measurement Techniques and Persistence

**DOI:** 10.3389/fpsyg.2016.01141

**Published:** 2016-08-03

**Authors:** Andre Kuijsters, Judith Redi, Boris de Ruyter, Ingrid Heynderickx

**Affiliations:** ^1^Human-Technology Interaction Group, Department of Industrial Engineering and Innovation Sciences, Eindhoven University of TechnologyEindhoven, Netherlands; ^2^Multimedia Computing Group, Department of Intelligent Systems, Delft University of TechnologyDelft, Netherlands; ^3^Distributed and Interactive Systems Group, Centrum Wiskunde en InformaticaAmsterdam, Netherlands; ^4^Brain, Cognition and Perception Group, Philips ResearchEindhoven, Netherlands

**Keywords:** sadness, anxiousness, MIP, mood, IAPS, persistence

## Abstract

The persistence of negative moods (sadness and anxiousness) induced by three visual Mood Induction Procedures (MIP) was investigated. The evolution of the mood after the MIP was monitored for a period of 8 min with the Self-Assessment Manikin (SAM; every 2 min) and with recordings of skin conductance level (SCL) and electrocardiography (ECG). The SAM pleasure ratings showed that short and longer film fragments were effective in inducing a longer lasting negative mood, whereas the negative mood induced by the IAPS slideshow was short lived. The induced arousal during the anxious MIPs diminished quickly after the mood induction; nevertheless, the SCL data suggest longer lasting arousal effects for both movies. The decay of the induced mood follows a logarithmic function; diminishing quickly in the first minutes, thereafter returning slowly back to baseline. These results reveal that caution is needed when investigating the effects of the induced mood on a task or the effect of interventions on induced moods, because the induced mood diminishes quickly after the mood induction.

## Introduction

Mood Induction Procedures (MIPs) are designed to experimentally control the affective state of participants to investigate the influence on cognitive, social, and neural processes (Westermann et al., 1996). A large variety of procedures exists, e.g., based on music listening (Västfjäll, 2002), viewing affectively charged videos (Gross and Levenson, 1995) or pictures (Lang et al., 2008), following instructions to imagine scenes (Velten, 1968) and recalling past events (Strack et al., 1985). MIPs are often used in experiments to control the mood of participants in order to test the effect of specific mood states on, amongst others, memory (Bower, 1981), creativity (Davis, 2009), neural processes (Mayberg et al., 1999), decision making (Isen and Means, 1983), behavior (Gendolla, 2000), and to test the effectiveness of affective interventions (Kuijsters et al., 2015). These experiments assume the induced mood to last until the entire experimental task (or intervention) has been carried out. It is therefore necessary that the mood induced by the MIP is long lasting and stable through time. In addition, mood is often distinguished from emotion by psychologists because of its longer-lasting nature (Scherer et al., 2001). If the object of investigation is mood, and not a temporary emotional state, it is then important to ensure that the MIPs induce more than instantaneous affective states.

Despite the remarkable body of work providing evidence for the effectiveness of the MIPs (Martin, 1990; Gerrards-Hesse et al., 1994; Westermann et al., 1996), little is known about how the induced moods evolve in time. The effectiveness of the MIPs is typically tested by comparing (subjective, self-reported) mood ratings before and after the MIP. A significant difference in mood ratings (mostly valence and/or arousal) is considered as an indication of the successful induction, but hardly any quantification of its persistence has been described in literature. In this study, we investigate the persistence of induced negative mood states, and the evolution of the mood after the mood induction.

### Persistence of the Induced Mood

The few studies that investigated the persistence of the induced moods reported mixed results. The moods induced with the Velten method (Velten, 1968), were not evident after an intervening task (Isen and Gorgoglione, 1983) or a 10 min waiting period (Frost and Green, 1982). Other studies reported that the induced valence was still salient after an intervening task (Chou et al., 2007; Kliegel et al., 2007; Gomez et al., 2009), while the induced arousal disappeared quickly (Gomez et al., 2009) when using self-selected film segments. The persistence of induced moods with a slideshow of affective pictures was investigated only for short periods of less than 60 s (Bradley et al., 1996; Smith et al., 2005).

If an induced mood persists after the affective stimulation, one may expect to observe this also in physiological responses of the participants, whose connection with affective states has been widely researched (Bradley and Lang, 2000; Cacioppo et al., 2000; Mauss and Robinson, 2009). Literature on the persistence of cardiac and electro dermal response after the affective stimulation is rare. Smith et al. (2005) found that the heart rate (HR) decelerates during viewing negative affective pictures, increases after exposure, and then remains elevated during a 30-s interval time. They argue that this rebound effect may be caused by mental processing after exposure, as HR increases during aversive imagery (Vrana and Lang, 1990). Similar results were found by Von Leupoldt and Dahme (2005), for both pleasant and unpleasant IAPS pictures. Others showed that the HR effect induced by visual exposure to affective stimuli disappears quickly after the exposure (Bradley et al., 1996), or is masked by the following task exposure (Gendolla and Krüsken, 2001; Gomez et al., 2009). Campbell-Sills et al. (2006) found that skin conductance level (SCL) increases while watching an anxious movie and remained elevated after a 2 min waiting period. Bradley and Lang (2000) found similar patterns of skin conductance responses during picture exposure and inter exposure periods; they were able to discriminate between low and high arousing affective pictures during exposure, but also after exposure. Gomez et al. (2009) found the induced skin conductance responses to diminish after visual exposure and to be more affected by the task after exposure than by the affective movies.

### Scope of the Paper

In general, literature indicates that the induced mood will diminish after the mood induction, returning, on average, to the mood the subject was in before the mood induction (baseline mood). Because in these studies mood was measured at best three times (i.e., before the MIP, after the MIP, and after the completion of a task/waiting period) it is still unknown for how long the induced mood persists and how the mood will return back to baseline. In addition, some of these studies did not use standardized mood induction material, but rather self-selected movie excerpts.

The goal of this study is to investigate the persistence of negative moods induced through different MIPs, and analyze the evolution of mood back to baseline after the MIP. We compare the duration of two negative moods, namely sadness and anxiety, induced by three different MIPs: a slide show of IAPS images (Lang et al., 2008), the viewing of short, standardized excerpts of affective videos (Gross and Levenson, 1995), and the viewing of longer excerpts of affective videos used in previous research intended to induce longer lasting moods (Kliegel et al., 2005, 2007; Gomez et al., 2009). These MIPs can effectively induce the targeted moods without the need for experimenter guidance or explicit instructions to try to mimic the mood (Westermann et al., 1996). The mood of the participant is assessed through self-assessment with the Self-Assessment Manikin (SAM; Bradley and Lang, 1994) before and after the MIP, and at regular intervals for the 8 min following the MIP. During the experiment, the mood is also monitored with physiological measures, and specifically with SCL and electrocardiography (ECG) signals.

## Materials and Methods

In this experiment, we monitored the evolution through time of the mood by three different MIPs: (1) viewing shorter standardized film segments, (2) viewing longer film segments, and (3) viewing an IAPS slides presentation. We were specifically interested in checking how long the induced mood would last per procedure, and which of the three would produce longer lasting effects. We limited our investigation to the induction of two negative mood states: a low arousing negative mood state (hereafter referred to as ‘sadness’) and a high arousing negative mood state (hereafter referred to as ‘anxiousness’). The two moods were investigated between subjects, i.e., two separate groups of participants underwent the induction of sadness and anxiety, respectively. The MIPs were instead tested within subjects, i.e., each participant (in both groups) underwent all three MIPs, in three separate sessions.

### Participants

Thirty participants took part in the experiment, and were randomly assigned to the ‘sadness’ or ‘anxiousness’ mood group, each group consisting of eight males and seven females. The participants were students and staff of the Delft University of Technology or the Eindhoven University of Technology. Mean age of the sample was 22.4 (*SD* = 3.03 range = 18–30)^1^.

### Mood Induction Procedures

#### IAPS Slides

We created two slideshows, consisting of twenty slides each, from the IAPS database (Lang et al., 2008). Each slide was presented for 5 s with a total presentation time of 1’40”. We used the normative ratings on sadness and fear (Libkuman et al., 2007) and the SAM pleasure and arousal ratings (Lang et al., 2008) to select the appropriate slides. For the sadness slideshow we selected images that were scored high on sadness and low on arousal and pleasure^2^. The average ratings (scale from 1 to 9) were 2.5 for pleasure, 4.7 for arousal, and 6.8 for sadness. For the anxiousness^3^ slideshow we selected images that scored high on fear, low on pleasure and high on arousal. The average ratings were 2.5 for pleasure, 6.7 for arousal, and 6.1 for fear. By creating a slideshow of pictures with similar affective connotation, the intended mood can be induced into the slideshow viewers (Bradley et al., 1996; Smith et al., 2005).

#### Short Standardized Film Fragments

Two short film fragments were selected from the database of Gross and Levenson (1995). For inducing sadness, the scene at which a boy cries at his father’s death (2’51”) from the movie ‘The Champ’ (Zeffirelli and Lovell, 1979) was selected. For inducing anxiousness, the basement chase scene (3’29”) from the movie ‘The silence of the lambs’ (Demme et al., 1991) was selected. Although the film fragments in the database of Gross and Levenson were designed for emotion elicitation, they are widely and successfully used to induce related mood states (see amongst others: Marzillier and Davey, 2005; Macht and Mueller, 2007; Converse et al., 2008).

#### Longer Film Fragments

Two film fragments with a length about three times as long as the ones present in the database of Gross and Levenson were selected. These film segments were chosen as they were employed in previous studies in which the researchers intended to induce longer lasting moods. The first fragment used the Krakow ghetto scene (10’08”) from the movie “Schindler’s List” (Spielberg et al., 1993) to induce sadness; this film segment was successfully used by Kliegel et al. (2005, 2007) to induce a sad mood state. The second fragment from the movie “The Deer Hunter” (Cimino et al., 1978) depicted captives forced to play Russian roulette (9’41”) and was used to induce anxiousness. This film segment was successfully used by Gomez et al. (2009) to induce an anxious mood state.

### Mood Measurements

The participants assessed their actual mood state with two questionnaires: the SAM (Bradley and Lang, 1994) and the pleasure–arousal–dominance questionnaire (PAD; Mehrabian and Russell, 1974). SAM was used to assess Pleasure and Arousal before the MIP, and at regular intervals of 2 min after the MIP. PAD was only used at the beginning and end of the experiment as control for the SAM. Only the SAM questionnaire was used for the regular assessment of mood during the mood recovery period because this well validated questionnaire is quick and low on cognitive effort for the participants. In addition, very high correlations are reported between SAM and the more elaborated PAD questionnaire (Bradley and Lang, 1994). However, these correlations are based on the evaluation of affective stimuli, and not on the evaluation of the participants’ own affective state. Therefore, we wanted to include the calibration of SAM against PAD at the beginning and end of the experiment.

In our experiment, we complemented mood self-reports with physiological measurements. We focused on unobtrusive measurements that can be performed on the hand and wrist of the participants; in particular, we used the electro dermal activity and cardiac response, which have been found to be related to mood changes. Increased electro dermal activity indicates increased physiological arousal, and this can be measured most robustly with the SCL (Lang et al., 1993; Bradley et al., 2001; Gomez et al., 2004, 2009; Kreibig et al., 2007). An increase in the cardiac activation [e.g., increased HR, or decreased heart rate variability (HRV)] also indicates increased physiological arousal (Task Force, 1996; Kreibig et al., 2007). Increased HRV is an indicator of an increased parasympathetic activity (i.e., resting state) and/or decreased sympathetic activity (i.e., active state; Task Force, 1996), where especially the high frequency component of HRV has been linked to parasympathetic activity (Task Force, 1996). HR is also linked to subjective pleasure ratings; it decelerates when watching negative visual stimuli (Bradley et al., 2001; Gomez et al., 2005). According to the defense cascade model (Lang et al., 1997) sustained cardiac deceleration can occur when continuously watching aversive stimuli, however action is not imminent. According to this model, people show physiological signs of heightened attention toward negative stimuli in absence of a highly relevant threat, namely HR deceleration and moderate skin conductance responses. While people prepare for immediate action if they are presented with highly relevant unpleasant stimuli (related to acceleration of the HR and larger skin conductance responses).

We measured physiological responses with the Nexus-10 measuring system (Mind Media BV, Roermond, The Netherlands)^4^. The device was controlled with the Biotrace software suite (Mind Media BV, Roermond, The Netherlands) via a computer. The SCL was recorded with the Nexus-10 SC/GSR sensors. The sensors were applied to the upper phalanxes of the index and middle finger of the non-dominant hand. For the ECG recordings, pre-gelled silver chloride electrodes were placed at the inside of the wrists of both arms, a third electrode was placed at the upper arm and served as ground sensor. For SCL, we had a saving rate of 32 Hz with a resolution of 0.001 μS; for ECG, the saving rate was 2048 Hz with a resolution of 1 μV.

### Procedure and Design

Participants were not informed about the specific goal of the experiment. They were told that the experiment investigated their physiological reactions to films and pictures. All participants were tested individually under controlled and uniform lighting settings. This study was approved by the institutional review board of the Eindhoven University of Technology and Delft University of Technology, Netherlands, in 2013; both boards followed the Code of Ethics of the Dutch Institute for Psychologists. All participants gave informed consent in accordance with the Declaration of Helsinki.

The three different MIPs were tested in three sessions, with at least 1 day between sessions. The order of the three MIPs was randomized in a different way for each participant. In each session, the procedure, identical for all participants, was controlled by a computer. To start, participants were welcomed and seated in a comfortable armchair placed 0.4 m from a 23 inch computer screen. They were then asked to wear headphones. Participants received information about the experimental layout, were explained the procedure and asked to sign the informed consent form. The sensors for the physiological measurements were then attached and the recording started; after a 5 min waiting period the baseline mood measurement was then recorded by means of the PAD and SAM (i.e., SAM1 hereafter) questionnaires. After the baseline mood measurement, the chosen MIP for that session was displayed on the computer screen, followed by a second SAM measurement (i.e., SAM2). Thereafter, the participants were requested to indicate their actual mood every 2 min with the SAM scales, for a total of 4 more mood measurements (i.e., SAM3 – SAM6), as also depicted in the scheme of **Table 1**.

**Table 1:** Scheme of the experimental procedure.

	Physiological measurements											
	
Preparations physiological measures	5 min	Baseline Mood: SAM1 and PAD1	MIP	Mood after MIP: SAM2	2 min	Mood 2 min: SAM3	2 min	Mood 4 min: SAM4	2 min	Mood 6 min: SAM5	2 min	Mood 8 min: SAM6 and PAD2


During the periods between the additional SAM measurements, visual kaleidoscope effects were presented on the screen for 2 min. These effects were chosen as neutral task to occupy the participants during the waiting periods, because a pilot test showed that participants were bored and annoyed by the waiting time if no stimulus was provided. Obviously, the annoyance could affect the measured mood. Roughly 8 min after the MIP participants also filled out the PAD questionnaire, which completed the session. After the third session the participants were debriefed and received a small gift as appreciation for their participation.

### Analysis

The recorded physiological measurements were processed in the following way. HR and HRV were computed offline from the ECG recordings by analyzing the variability in the intervals between sinus rhythm heart beats (R–R intervals) following the guidelines of the task force of the European Society of Cardiology and the North-American Society of Pacing and Electrophysiology (Task Force, 1996). Custom software was written in MATLAB for the R-wave detection; the complete signal was carefully visually inspected and false or undetected R-waves, ectopic beat errors, and movement artifacts were manually corrected. HR (beats per minute) was calculated from the inter-beat-intervals. Time and frequency domain HRV measures were analyzed using KARDIA (Perakakis et al., 2010) in MATLAB. The RMSSD (the square root of the mean squared differences of successive R–R intervals) was calculated as indicator of short term HRV. The High Frequency component (HF-HRV; 0.15–0.40 Hz) of HRV was calculated in the frequency domain with Fast Fourier Transform analysis (resample rate = 2 Hz, FFT window length = 512). The HF-HRV was considered as a specific indicator of parasympathetic activity, expressed in absolute values of power (ms^2^).

For all the physiological indicators and for every participant, *change values* were calculated in correspondence of the six times of SAM measurement. The median signal values of the last 100 s of the baseline were subtracted from the median values of the last 100 s of the mood induction period, and of any following waiting period. A buffer of 10 s was applied to avoid time-alignment errors. These change values served as dependent variables for further analysis. For the self-reported SAM measures we resorted to what we call “Affective change scores.” Affective change scores were calculated for every mood measurement moment after the baseline measurement and for every participant by subtracting the affective rating at the end of the baseline (i.e., SAM1) from the affective rating measured after the mood induction and the four waiting periods (i.e., SAM2 – SAM6).

The normality assumption for parametric statistical analysis was violated; the data was highly skewed. Therefore we analyzed our data with the non-parametric Friedman test. These tests were carried out to investigate a main effect of time (i.e., at the six specific mood measurement moments) on both the pleasure and arousal scores for each MIP. Similar Friedman tests were carried out to investigate a main effect of time on the physiological change values for each MIP. The significance level was set at α = 0.05 for all Friedman tests. With the Wilcoxon *post hoc* test we compared the baseline scores with the five other moments for each MIP; Bonferroni correction was applied resulting in a significance level of 0.05/5 = 0.010.

The evolution of the mood after the mood induction was further investigated with regression analysis. In the analyses, time (*X*) is the predictor of the individual pleasure and arousal change scores (*Y*) for each MIP, separately for the sad and anxious mood group. *X* = {1, 3, 5, 7, 9} represented the measurement moments (Mood induction, 2 min, 4 min, 6 min, 8 min, respectively). We first investigated whether the relation between time and the affective change sscores was linear or not. A linear model (*Ŷ* = *a* + *bX*) wa compared with a logarithmic (*Ŷ* = *a* + *b*log *X*) and a quadratic model (*Ŷ* = *a* + *bX* + *cX*^2^). The decision on the best model was based on the explained variance (*r*^2^) and the root-mean-square error (RMSE) of the models: the more complex quadratic model would only be preferred if adding the quadratic component in the function would decrease the RMSE and explain significantly more variance compared to the simpler linear and logarithmic models. We also investigated if the evolution of the mood was different between the three MIPs; i.e., if the model coefficients were significantly different between the MIPs. Bonferroni correction was applied for the pairwise comparison of the three MIPs, resulting in a significance level of 0.05/3 = 0.017.

## Results

### Anxious Mood Induction – Effect on SAM Ratings

**FIGURE 1:**
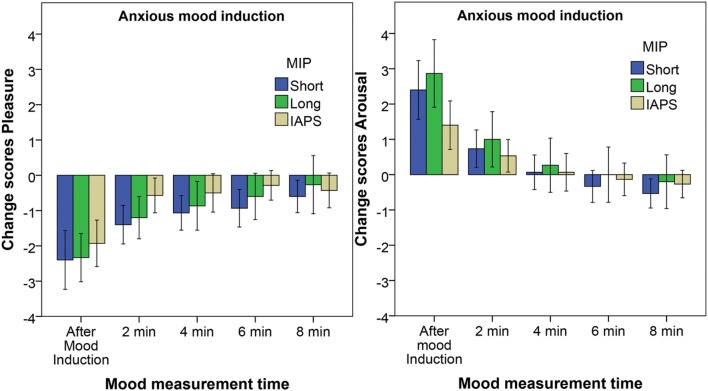
**Pleasure change scores **(Left)** and arousal change scores **(Right)** for the Anxious mood group.** The different bars reflect the three different MIPs. Error bars reflect the 95% confidence interval.

**Figure 1** shows the affective change scores for each of the five mood measurement moments for the Anxious mood group; the different bars depict the three different MIPs with the error bars reflecting the 95% confidence interval. The Friedman test reveals a significant effect of time on the pleasure change score for all three MIPs: Short film fragment *X*^2^(5) = 43.6, *p* < 0.001, Long film fragment *X*^2^(5) = 29.3, *p* < 0.001, and IAPS slides *X*^2^(5) = 27.4, *p* < 0.001. The results of the Wilcoxon *post hoc* tests are summarized in **Table 2**. They reveal that for all three MIPs the pleasure scores are significantly lower after the mood induction compared to the baseline measurement. For the Short film fragment the pleasure change scores remain significantly below the baseline up to and including the mood measurement after 6 min. For the Long film fragment the pleasure change scores are significantly below the baseline for all moments except for the measurement at 4 min after the MIP. For the IAPS slide show the pleasure scores remain significantly below the baseline up to and including the measurement 2 min after the MIP.

**Table 2:** Results of the Wilcoxon *post hoc* tests for the pleasure and arousal scores of the Anxious mood group with respect to baseline.

		Mood Induction	2 min	4 min	6 min	8 min
**Pleasure**	Short film fragment	*Z* = 3.47	*Z* = 3.04	*Z* = 3.21	*Z* = 2.97	*Z* = 2.27
		*p* = 0.001	*p* = 0.002	*p* = 0.001	*p* = 0.003	*p* = n.s.
		*r* = 0.63	*r* = 0.55	*r* = 0.59	*r* = 0.54	
	Long film fragment	*Z* = 3.19	*Z* = 2.85	*Z* = 2.49	*Z* = 2.95	*Z* = 2.99
		*p* = 0.001	*p* = 0.004,	*p* = n.s.	*p* = 0.003	*p* = 0.003,
		*r* = 0.58	*r* = 0.52		*r* = 0.54	*r* = 0.55
	IAPS slideshow	*Z* = 3.11	*Z* = 2.83	*Z* = 0.91	*Z* = 1.43	*Z* = 1.80
		*p* = 0.001	*p* = 0.005	*p* = n.s.	*p* = n.s.	*p* = n.s.
		*r* = 0.57	*r* = 0.52			
**Arousal**	Short film fragment	*Z* = 3.45	*Z* = 2.48	*Z* = 0.30	*Z* = 1.52	*Z* = 2.27
		*p* = 0.001	*p* = n.s.	*p* = n.s.	*p* = n.s.	*p* = n.s.
		*r* = 0.63				
	Long film fragment	*Z* = 3.34	*Z* = 2.25	*Z* = 0.54	*Z* = 0.000	*Z* = 0.425
		*p* = 0.001	*p* = n.s.	*p* = n.s.	*p* = n.s.	*p* = n.s.
		*r* = 0.61				
	IAPS slideshow	*Z* = 3.134	*Z* = 2.126	*Z* = 0.277	*Z* = 0.632	*Z* = 1.414
		*p* = 0.002	*p* = n.s.	*p* = n.s.	*p* = n.s.	*p* = n.s.
		*r* = 0.57				


For the arousal change scores a significant effect of time is found for the three MIPs: Short film fragment *X*^2^(5) = 53.882, *p* < 0.001, Long film fragment *X*^2^(5) = 46.1, *p* < 0.001, and IAPS slides *X*^2^(5) = 27,961, *p* < 0.001. The Wilcoxon *post hoc* tests (also summarized in **Table 2**) reveal that the arousal scores are significantly higher after the mood induction for all three MIPs. The arousal change scores, however, are not significantly different from baseline anymore at and after the measurement of 2 min after the MIP.

### Anxious Mood Induction – Effect on Physiological Measures

**FIGURE 2:**
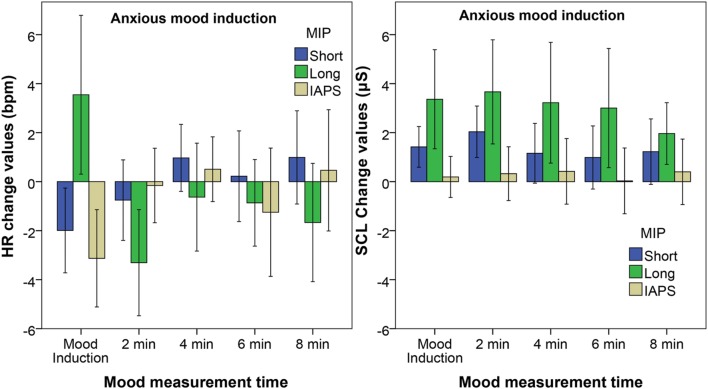
**Adjusted means of the change values of heart rate **(Left)** and skin conductance **(Right)** at the end of the five measurement moments relative to the baseline for the Anxious mood group.** The different bars represent the different MIPs with the error bars reflecting the 95% confidence interval.

**Figure 2** shows the change values for HR and skin conductance response for the Anxious mood group. The HR accelerates during mood induction with the Long film fragment, while it subsequently decelerates during the first waiting period of 2 min. For the mood induced by the Short film fragment and the IAPS slides the HR decelerates during mood induction. The Friedman test reveals a significant effect of time on the HR for the all three MIPs: Short film fragment *X*^2^(5) = 16.295, *p* = 0.006, Long film fragment *X*^2^(5) = 17.743, *p* = 0.003, and IAPS slides *X*^2^(5) = 13.265, *p* = 0.021. The Wilcoxon *post hoc* tests, however, reveal no significant differences in change values across the different mood measurement times for the Short film fragment, when the Bonferroni correction is applied. For the Long film fragment only the measurement after 2 min is significantly different from baseline: *Z* = 2.669, *p* = 0.008, *r* = 0.49. For the IAPS slides, instead, the HR significantly decelerates during the mood induction: *Z* = 2.67, *p* = 0.008, *r* = 0.49.

A significant effect of time on SCL is found for the Short film fragment *X*^2^(5) = 16.2, *p* = 0.006 and Long film fragment *X*^2^(5) = 25.8, *p* < 0.001, however not for the IAPS slide show. The *post hoc* tests reveal that the SCL increases significantly during mood induction: for the Short film fragment *Z* = 2.95, *p* = 0.003, *r* = 0.54, and for the Long film fragment *Z* = 3.23, *p* = 0.001, *r* = 0.59. For the Short film fragment also the measurement after 2 min is significantly higher than baseline *Z* = 3.181, *p* = 0.001, *r* = 0.58, while for the Long film fragment all mood measurement moments are significantly higher than baseline; ‘2 min’ *Z* = 3.11, *p* = 0.002, *r* = 0.56, ‘4 min’ *Z* = 3.11, *p* = 0.002, *r* = 0.57, ‘6 min’ *Z* = 3.17, *p* = 0.002, *r* = 0.58, and ‘8 min’ *Z* = 2.73, *p* = 0.006, *r* = 0.50.

For the RMSSD a significant effect of time is found only for the IAPS slide show *X*^2^(5) = 12.667, *p* = 0.027. In this case, the *post hoc* tests, however, reveal no significant differences between the different measurement moments, when the Bonferroni correction is applied. The effect of time on HRV-HF is also only significant for the IAPS slide show *X*^2^(5) = 14.000, *p* = 0.016, but again here no significant differences are found with the *post hoc* test.

### Sad Mood Induction – Effect on SAM Ratings

For the Sad mood group a significant effect of time on the pleasure change scores is found for all three MIPs: Short film fragment *X*^2^(5) = 43.9, *p* < 0.001, Long film fragment *X*^2^(5) = 46.8, *p* < 0.001, and IAPS slides *X*^2^(5) = 36.0, *p* < 0.001. The actual affective change scores and the results of the Wilcoxon *post hoc* tests are summarized in **Table 3**. Again the pleasure change scores significantly decreases after the mood induction for all three MIPs. For the Short film fragment, the pleasure change scores remain significantly below the baseline up to and including the measurement after 6 min, as can be deduced from **Table 3**. For the Long film fragment the pleasure scores are significantly below the baseline scores only up to and including the measurement after 2 min. Lastly, for the IAPS slide show the pleasure scores are not significantly below baseline anymore at 2 min after mood induction.

**Table 3:** Results of the Wilcoxon *post hoc* test for the pleasure and arousal scores of the Sad mood group with respect to baseline.

		Mood Induction	2 min	4 min	6 min	8 min
**Pleasure**	Short film fragment	*Z* = 3.454	*Z* = 3.439	*Z* = 2.889	*Z* = 2.640	*Z* = 2.310
		*p* = 0.001	*p* = 0.001	*p* = 0.004	*p* = 0.008	*p* = n.s.
		*r* = 0.63	*r* = 0.63	*r* = 0.53	*r* = 0.48	
	Long film fragment	*Z* = 3.319	*Z* = 2.886	*Z* = 2.131	*Z* = 1.812	*Z* = 0.784
		*p* = 0.001	*p* = 0.004	*p* = n.s.	*p* = n.s.	*p* = n.s.
		*r* = 0.63	*r* = 0.53			
	IAPS slideshow	*Z* = 3.213	*Z* = 2.126	*Z* = 1.897	*Z* = 1.414	*Z* = 1.732
		*p* = 0.001	*p* = n.s.	*p* = n.s.	*p* = n.s.	*p* = n.s.
		*r* = 0.59				
**Arousal**	Short film fragment	*Z* = 1.403	*Z* = 0.642	*Z* = 0.773	*Z* = 0.392	*Z* = 0.476
		*p* = n.s.	*p* = n.s.	*p* = n.s.	*p* = n.s.	*p* = n.s.
	Long film fragment	*Z* = 2.654	*Z* = 1.268	*Z* = 0.106	*Z* = 1.316	*Z* = 1.812
		*p* = 0.008	*p* = n.s.	*p* = n.s.	*p* = n.s.	*p* = n.s.
		*r* = 0.48				
	IAPS slideshow	*Z* = 2.032	*Z* = 0.694	*Z* = 0.142	*Z* = 0.575	*Z* = 0.979
		*p* = n.s.	*p* = n.s.	*p* = n.s.	*p* = n.s.	*p* = n.s.


**FIGURE 3:**
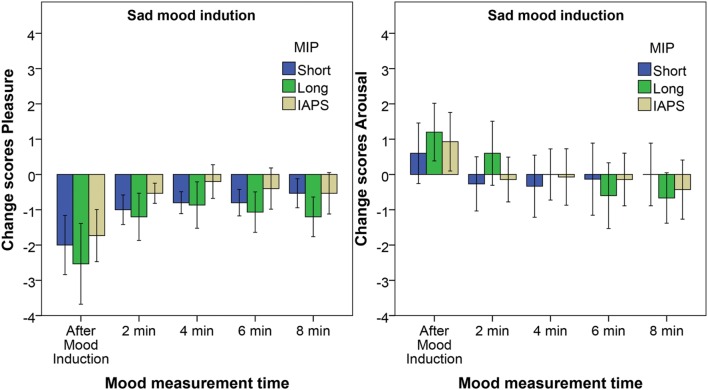
**Pleasure change scores **(Left)** and arousal change scores **(Right)** for the Sad mood induction.** The different bars reflect the three different MIPs. Error bars reflect the 95% confidence interval.

For the arousal change scores a significant effect of time is found for the three MIPs: Short film fragment *X*^2^(5) = 12.7, *p* = 0.027, Long film fragment *X*^2^(5) = 27.2, *p* < 0.001 and IAPS slides *X*^2^(5) = 12.1, *p* = 0.033. *Post hoc* tests reveal that the arousal change scores are significantly higher than baseline after the mood induction only for the Long film fragment. All other differences are not significant when the Bonferroni correction is applied (**Figure 3**).

### Sad Mood Induction – Effect on Physiological Measures

**FIGURE 4:**
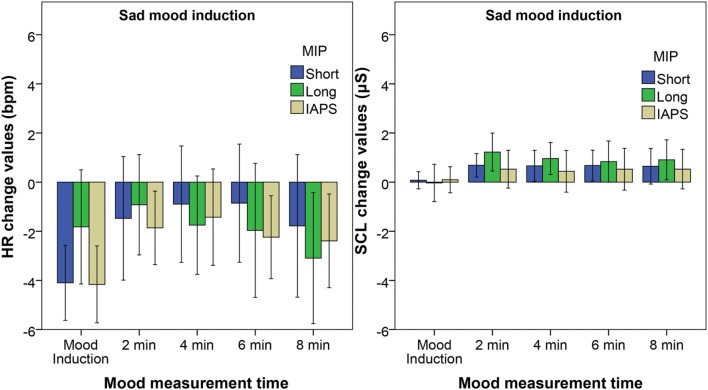
**Adjusted means of the change values of heart rate **(Left)** and skin conductance **(Right)** at the end of the five measurement moments relative to the baseline for the Sad mood group.** The different bars represent the different MIPs with the error bars reflecting the 95% confidence interval.

The change values for HR and SCL for the Sad mood group are displayed in **Figure 4**. The HR decreases during mood induction with the Short film fragment and the IAPS slides. The Friedman tests reveal a significant effect of time on the HR for indeed the Short film fragment *X*^2^(5) = 18.6, *p* = 0.002 and the IAPS slides *X*^2^(5) = 25.1, *p* < 0.001. The Wilcoxon *post hoc* tests reveal that the HR decelerates during the mood induction for the Short film fragment *Z* = 3.35, *p* = 0.001, *r* = 0.61 and the IAPS slides *Z* = 3.30, *p* = 0.001, *r* = 0.60. All other differences are not significant.

For the SCL a significant effect of time is found with both the Short film fragment *X*^2^(5) = 12.0, *p* = 0.034 and the Long film fragment *X*^2^(5) = 21.5, *p* = 0.001. However the *post hoc* tests reveal no significant differences for the Short film fragment after Bonferroni correction. For the Long film fragment the SCL is significantly above baseline after 2 min *Z* = 2.78, *p* = 0.005, *r* = 0.51 and 4 min *Z* = 2.67, *p* = 0.008, *r* = 0.48. No significant effect of time is found on the RMSSD or HRV-HF for all three MIPs.

### Evolution of the Induced Mood

Regression analysis reveals that the decay of the induced mood (as of after the mood induction) can be best described by a logarithmic function. The logarithmic models, in which the log(time) predicts the affective change scores of both pleasure and arousal after the mood induction, explain more variance and had a lower RMSE compared to the linear models, as reported in **Table 4**. Adding a quadratic component to the regression function does not significantly improve the models’ accuracy, and therefore these results are not reported.

**Table 4:** Summary of the regression analyses for the pleasure and arousal change scores after the mood induction.

		MIP	Slope	Intercept	*F*(1,73)	*p*	*r*^2^	*RMSE*	*r*^2^ linear	*RMSE* linear
**Anxious mood group**	Pleasure	Short	0.635	-1.90	22.3	<0.001	0.23	0.892	0.19	0.918
		Long	0.656	-2.27	10.5	0.002	0.13	1.35	0.08	1.38
		IAPS	0.596	-1.50	15.4	<0.001	0.17	1.01	0.10	1.05
	Arousal	Short	-1.38	2.33	83.5	<0.001	0.53	0.988	0.46	1.06
		Long	-1.42	2.74	43.8	<0.001	0.38	1.43	0.31	1.50
		IAPS	-0.775	1.38	32.6	<0.001	0.31	0.902	0.27	0.926
**Sad mood group**	Pleasure	Short	0.787	-2.36	26.0	<0.001	0.26	1.023	0.23	1.05
		Long	0.909	-2.30	24.8	<0.001	0.25	1.21	0.23	1.23
		IAPS	0.769	-1.85	27.6	<0.001	0.30	0.911	0.19	0.958
	Arousal	Short	-0.300	0.384	1.62	0.208	0.02	1.57	0.01	1.58
		Long	-0.894	1.33	16.9	<0.001	0.19	1.45	0.19	1.45
		IAPS	-0.558	0.793	7.55	0.008	0.10	1.30	0.08	1.32


*The predictor variable was the logarithmic of time (log *X*). The last columns displays the *r*^*2*^ and the root-mean-square error (RMSE) of the linear model with time (*X*) as predictor variable.*

**FIGURE 5:**
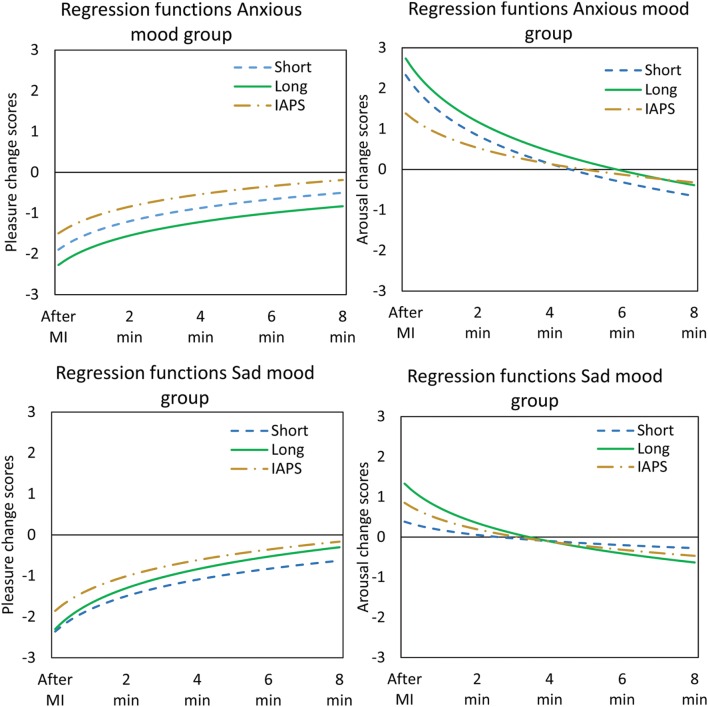
**The regression functions for the pleasure change scores **(Left)** and arousal change scores **(Right)** separate for the anxious mood group **(Above)** and sad mood group **(Under)**.** The different lines represent the different MIPs.

The coefficients of the logarithmic models are also displayed in **Table 4**. The intercept can be regarded as the affective change score for pleasure or arousal as a result of the mood induction. The regression functions are plotted in **Figure 5**.

To test for significant differences between the evolutions over time of the three MIPs we pool the data for the sad mood group and for the anxious mood group. MIP and the interaction MIP^∗^log(time) were stepwise included in logarithmic regression equations as predictors. The MIP^∗^log(time) interaction term compares the regression coefficients (slope) among the three MIPs.

A significant effect of MIP is found for the pleasure change scores after anxious mood induction *F*(2,221) = 7.323, *p* = 0.001. Only the Long film fragment and the IAPS slide show are significantly different *t* = -3.83, *p* < 0.001; more displeasure is induced by the Long film fragment and this difference is maintained over time (see **Figure 5**). The slope coefficients are not significantly different among the three MIPs. Also a significant effect of MIP is found for the arousal change scores *F*(2,221) = 3.16, *p* = 0.044. Again only the Long film fragment and the IAPS slide show are significantly different *t* = -2.46, *p* = 0.015; more arousal is induced by the Long film fragment compared to the IAPS slide show. The regression coefficients are significantly different across the three MIPs *F*(2,221) = 4.40, *p* = 0.001. The slope of the curve fitting for the IAPS slide show is significantly less negative compared to that corresponding to the Short film fragment *b* = 0.582, *t* = 2.42, *p* = 0.016 and the Long film fragment *b* = 0.647, *t* = 2.69, *p* = 0.008; the intercept of the IAPS slide show is significantly lower compared to both the intercept of the Short film fragment *b* = -0.944, *t* = -2.49, *p* = 0.013 and the Long film fragment *b* = -1.35, *t* = -3.57, *p* < 0.001.

After the sad mood induction a significant effect of MIP *F*(2,216) = 4.57, *p* = 0.011 is found for the pleasure change scores. Only the Short film fragment and the IAPS slide show are significantly different *t* = -3.02, *p* = 0.003; more displeasure is induced by the Short film fragment compared to the IAPS slide show and this difference is maintained over time (**Figure 5**). The regression coefficients are not significantly different among the three MIPs. For the arousal change scores, no significant effects are found on MIP or the regression coefficients.

### Correlation of SAM and PAD Ratings

Along with the SAM, the participants rated their mood at the start and at the end of the experiment with the PAD semantic differential scales. Spearman’s correlations are calculated between the PAD Pleasure scores and the corresponding SAM Pleasure scores, as measured at baseline and after 8 min. The same is done for Arousal. Scores are averaged across participants. The Pleasure scores of PAD and SAM are highly correlated *r*(12) = 0.84, *p* = 0.001. Also the arousal scores of PAD and SAM are highly correlated *r*(12) = 0.90, *p* < 0.001.

## Discussion

When aiming at inducing anxiousness, all three investigated MIPs were effective in inducing a high arousing negative mood state. Self-reported arousal significantly increased, while pleasure ratings were significantly reduced after the mood induction, with respect to baseline mood measurement. The pleasure ratings remained significantly below baseline for over 6 min for the movie-based MIPs, whereas the effect was shorter (only about 2 min) for the IAPS slide show. The regression analyses showed that the anxious movies induced more displeasure compared to the IAPS slide show and that this difference was maintained over time. However, only the difference between the Long film fragment and IAPS slide show was significant.

The self-reported arousal levels were not significantly different from the baseline ratings 2 min after the induction for all three MIPs. The regression analyses revealed that both movies induced significantly more arousal than the IAPS slide show; however, this dissipated quickly. In line with previous research, (self-reported) induced arousal seemed to decay quickly (Gomez et al., 2009). On the other hand, the electro-dermal activity suggested longer-lasting arousal effects. In our experiments, the significant increase in SCL when watching the anxious movies remained salient for about 2 min for the Short film fragment and even for more than 8 min for the Long film fragment. This result is in line with the work of Campbell-Sills et al. (2006) who found that the SCL increased during an anxious movie and remained elevated over a 2 min waiting period. They however did not measure mood over a longer time period. In contrast, the SCL remained stable around baseline when watching the IAPS slide show.

The analysis of cardiac response to anxiety induction revealed mixed results. The HR increased, as expected, while watching the anxious Long film fragment, however not significantly. Two minutes after mood induction the HR decreased to a level which was significantly below baseline. This may be a coping effect; the participants trying to relax after the anxious movie, thereby reducing their HR. After 4 min the HR had returned back to baseline. While watching the slide show the cardiac activation decreased. This result is in line with previous research indicating a deceleration of the HR when watching anxious slides (Ritz et al., 2005; Sokhadze, 2007). Bradley et al. (2001) argued that cardiac deceleration is the initial reaction to a threat stimulus; they revealed that the HR decelerates in the first 5 s of exposure. The anxious slide show is a combination of twenty different threatening images with an exposure time of 5 s which may explain the deceleration of the HR. The combination of our findings suggests, however, that viewing the slide show did not accumulate to an anxious mood state, which is expected to be accompanied with an increased cardiac response. No clear results were found on HR variability. Although we found main effects on RMSSD and HRV-HF, the Wilcoxon *post hoc* tests revealed no significant differences with respect to baseline when a Bonferroni correction was applied.

To induce sadness, the Short standardized film fragment seemed to be the most effective MIP. The self-reported pleasure ratings were significantly lower after viewing the sad Short fragment than before, and this difference remained salient for about 6 min; no significant differences in arousal were recorded instead, indicating that the initial low arousing state of the participants was unaltered. The Long film fragment was also effective in reducing the self-reported pleasure ratings; however, the induced negative state remained salient for only 2 min. Furthermore, the longer sad movie also induced self-reported arousal. This may be explained by taking a closer look at the content of the video chosen for the sad Long film fragment. It was an excerpt of Schindler’s list, specifically portraying the deportation of Jews from the Krakow ghetto during World War II; it is possible that the participants experienced mixed affective states: sadness due to the foreseen fate of the movie characters, but also fear for them. Thus, it is not unlikely that this movie also induced higher arousing negative affective states, such as anger or frustration. Finally, the IAPS slide show was also found to be effective in inducing a low arousing negative mood state; however, the self-reported pleasure and arousal ratings were no longer significantly different from baseline after 2 min. For the sad mood group, the regression analysis revealed only a significant difference between the Short film fragment and the IAPS slide show. The Short film fragment induced more displeasure compared to the IAPS slide show, and this difference remained salient over the 8 min after the mood induction.

In line with previous research, we found a decreased cardiac activity for the IAPS slide show and the Short film fragment, with the HR decelerating during the MIP. The deceleration of the HR did not persist after the mood induction, as the HR was not significantly different from baseline after 2 min. HR was not found to decrease while viewing the sad Long film Fragment, instead. This might be explained by the fact that this movie also induced some arousal (as found from significantly increased self-reported ratings), which may have dismissed the decrease in HR. The HRV measures did not reach significance for the three sad MIPs.

The electro dermal activity presented less clear results. The SCL was almost not affected during the three sad MIPs, but afterward the SCL increased (although only significantly so for the longer film fragment during the first 4 min). This might be possibly due to the induction of additional emotions when watching the longer sad movie, as discussed above. Higher arousing affective states, like anger, might sink in right after the MIP and persist for a while after it (although this finding was not backed by the self-reported ratings). Another explanation might be that the resting period observed before recording the baseline (set to 5 min in this experiment) was not long enough to ensure a stable baseline measurement for SCL. However, closer examination of the SCL signals revealed that the slope of the SCL response was close to zero for all three sad mood induction groups, which indicates that the SCL was stable during the baseline measurements.

The SAM arousal and valence scores were found to correlate well with the judgment of the verbal, semantic differential scales (PAD). This indicates that the quick and short SAM measurement can be used as an effective replacement of the longer semantic differential scales, also when used for affect self-reports. The correlations were not as high as reported by Bradley and Lang (1994). However, the latter correlations were based on the evaluation of affective stimuli, and not on the evaluation of the participants’ own affective state. Furthermore, the PAD was only scored at the beginning (baseline) and the end of the experiment. This resulted in a low variability among the observations, given that for most participants their mood returned back to their baseline mood at the end of the experiment. The correlation coefficient will be lower when there is less variability between the observations.

Although every experimental setup has its limitations, we did our utmost to make the most appropriate design choices, taking into account practical issues when inducing a negative mood state in people. Using participants over a broader range of age, culture, personality and background, for example in terms of movie knowledge, could shed more insight on individual differences in effectiveness of the MIPs and their evolution over time, but also would put a burden of a negative mood to a larger group of participants. These possible research questions are certainly interesting extensions of the current work, but should be chosen with care in view of the disadvantageous personal result of the experiment for the participants.

Within the research question we wanted to address, we made a number of design choices, and we would like to discuss them here. Firstly we choose three different MIPs; standardized movie excerpts, longer movies, and the IAPS slide show. The movie MIPs were chosen because movies are regarded as most effective MIP (Westermann et al., 1996) and they can be used without explicit instructions to get in a particular mood. The standardized movies from the database of Gross and Levenson (1995) are widely used in mood research and are available with editing instructions. The two longer movies were successfully used to induce longer lasting mood states in previous research (Kliegel et al., 2005, 2007; Gomez et al., 2009). The movies differ in duration, but we didn’t consider duration in itself as a factor for our research question; we were mainly interested in the evolution of the mood after the induction. The optimal length of a movie MIP though is a very interesting question for further research. The IAPS pictures are widely used in affective research and can induce short term mood states by creating a slide show of pictures with similar affective loadings (Bradley et al., 1996; Smith et al., 2005). If affective slides could induce mood states as effectively as movies this would open a world of new opportunities for mood research, because of the large freely available database of calibrated pictures (Lang et al., 2008). Our results however show that the IAPS slide show was less effective than the movies in inducing longer lasting moods.

Secondly, we decided to quantify the experienced mood at five different time instances with SAM and only at the beginning and end of the experimental session with PAD. As argued before, we motivated this choice by stressing that the mood measurement in itself should affect the natural reduction of the induced mood over time as least as possible. We already confirmed – from literature – the high correlation between SAM and PAD where we could compare them, and so we are inclined to only use SAM for our further research.

Thirdly, we decided to use kaleidoscope visuals during the waiting periods, as we considered them the best balance between not inducing mood by a task and inducing boredom by waiting without task. Pilot testing revealed that, without any task to be performed in the waiting period, participants were annoyed and bored by the four times 2 min of idleness. Kaleidoscope visuals were chosen as a neutral stimulus to keep the participants visually occupied, assuming these visuals would not influence the participants’ affective state. It might be the case, though, that the visuals reduced the arousal experienced by the participants. In future work the neutral stimulus or task should be more explicitly pretested on its affective response to minimize its affective influence.

Fourthly, we limited the physiological measurements to non-intrusive measurements on the hands and wrists. Unfortunately, these physiological measurements didn’t yield obvious confirmation of the trends in self-reported mood. In most cases, they didn’t exhibit a (statistically significant) change at all. In that respect, we acknowledge that timeframes of 100 s for the analysis of the HRV data may be relatively short. Most researchers recommend periods of 5 min for short term HRV recordings (Task Force, 1996). The longer recording time might result in clearer HRV data and the possibility to calculate normative values of HRV-HF and the HRV-HF/HRV-LF ratio. The setup of this experiment did not allow the selection of longer timeframes for the analysis of the physiological data, because we needed to measure self-reported mood every 2 min, given that previous research revealed that the induced mood can diminish rapidly (Frost and Green, 1982; Isen and Gorgoglione, 1983). The latter fact is confirmed with our results, since a logarithmic decay was the model best fitting our data. Thus, this suggests that HRV related measures are probably not very appropriate to measure decay in mood over time. It would have been interesting to investigate additional physiological signals in this setup, such as the ones that were found to be correlated with people’s affective state: corrugator EMG and respiration (Cacioppo et al., 2000). Using such signals might create a clearer picture of the physiological persistence effects of the induced moods, but these signals are also more intrusive, and so more prone to influence the affective state of the participants.

Lastly, we only induced negative affective states and no neutral or positive affective states. As a result, we cannot control for physiological reactions on watching visual stimuli, as also neutral film segments can induce physiological responses (Gomez et al., 2005). Including a neutral film segment might shed a clearer picture of the physiological persistence effects.

## Conclusion

We conclude mainly based on the self-reported mood data that short and longer movies are effective in inducing longer lasting moods, whereas this is less true for the IAPS slide show, for both sadness and anxiousness. Actually, our research suggests that a carefully selected short film clip can already be effective in inducing a longer lasting mood. In line with previous findings we found that induced valence is more persistent, while induced arousal diminishes quickly (Gomez et al., 2009). The results also reveal that evolution of the mood after the mood induction can be best described with a logarithmic function. The induced mood diminishes quickly in the first 2 min, thereafter returning slowly back to baseline. The physiological data we used didn’t fully confirm these findings, and more research is needed to evaluate whether a better implementation of these non-intrusive measures or the use of more intrusive measures is needed to confirm the self-reported mood physiologically. Our results also endorse that caution is needed when investigating the effect of task or intervention on induced mood states. Tasks should be relatively short or efforts are needed to re-induce or sustain the induced mood, for instance by playing affective music after the mood induction.

## Author Contributions

All author contributed to the development of the study hypothesis and study design. AK performed the data analysis and drafted the manuscript with input from JR, BdR, and IH. All authors performed critical revisions and approved the final version of the manuscript for submission.

## Conflict of Interest Statement

The authors declare that the research was conducted in the absence of any commercial or financial relationships that could be construed as a potential conflict of interest.

## Footnotes

1 Sample size was calculated *a priori* using G^∗^Power, based on a repeated measure ANOVA with three within groups (MIPs); given a power of 1 - β = 0.8 and an effect size of *f* = 0.3 (medium effect). Reported effect sizes for visual MIPs are very high (Westermann et al., 1996), however, unknown for persistence effects; only medium or greater effects are regarded to be meaningful.

2 IAPS images ‘sadness’ group: 2141, 2205, 2276, 2520, 2700, 2750, 2900, 3180, 3230, 3300, 3301, 9000, 9041, 9140, 9180, 9220, 9265, 9415, 9421, and 9561.

3 IAPS images ‘anxiousness’ group: 1050, 1052, 1300, 1302, 1931, 3000, 3053, 3064, 3530, 6230, 6250, 6321, 6350, 6550, 6560, 6570, 9410, 9620, 9911, and 9921.

4 http://www.mindmedia.info/CMS2014/nl/producten/systemen/nexus-10-mkii
